# Could bat-to-human spillover explain Ethiopia’s emerging Marburg virus cases

**DOI:** 10.1097/MS9.0000000000004863

**Published:** 2026-03-25

**Authors:** Naveed Ahmed Khan, Aaima Zain, Muaz Ahmed, Kamil Ahmad Kamil

**Affiliations:** a Department of Medicine, Khyber medical College, Peshawar, Pakistan; b Department of Medicine, Akhtar Saeed Medical & Dental College, Lahore, Pakistan; c Internal Medicine Department, Mirwais Regional Hospital, Kandahar, Afghanistan

Marburg virus disease (MVD), formerly known as Marburg hemorrhagic fever, causes a severe often fatal, viral hemorrhagic fever with a high case fatality rate^[^1^]^.It belongs to the same family as the Ebola virus and is transmitted to humans from fruit bats and then spreads through direct contact with the body fluids of infected people or animals^[^1,2^]^. Symptoms include fever, chills, and headache, and severe cases may progress to bleeding, shock, and organ failure^[^1^]^. The average case fatality rate is 50%, and the case reports have risen from 24% to 88% in the past outbreaks^[^1^]^. Early symptomatic treatment and rehydration improve survival^[^1^]^. Currently, no approved vaccines or antiviral treatments are available^[^1,2^]^.

Ethiopia’s ecological conditions, bat–human interactions, and limited access to rapid viral diagnostics in rural areas increase the risk of delayed detection and potentially widespread transmission. Ethiopia’s southern region has high bat–human interaction, limited diagnostic facilities, and delays in seeking formal care due to reliance on traditional medicine – factors that can slow early detection and increase the risk of Marburg virus spread. Additionally, population movement across porous borders further heightens the need for rapid containment.

Studies from South Africa have demonstrated that Egyptian rousette bats (ERB) actively shed Marburg virus RNA in their feces, confirming that environmental contamination of caves and feeding sites is a realistic source of human infection. Although Ethiopia lacks similar ecological studies, the presence of ERB populations in the affected region raises concerns that comparable shedding and environmental contamination may have facilitated the current outbreak^[^3^]^. These explanations could possibly explain the recent spillover of the Marburg virus in Ethiopia.

On November 12, the WHO noted a press release from the Ethiopian Ministry of Health and the Ethiopian Public Health Institute announcing a suspected viral fever in Jinka Town, South Ethiopian Regional State^[^4,5^]^. On November 14, it was confirmed that the suspected viral fever was actually MVD^[^4^]^, the first confirmed outbreak of MVD in the country^[^2,4–6^]^. Initially, six confirmed cases were reported, leading to three deaths^[^4^]^. Initial investigations in Ethiopia have shown the presence of the natural host of the virus, fruit bats, in the area^[^4^]^. Such presence causes “spillover” from bats to humans and further spread through human-to-human interaction. Patients present with high-grade fever, headache, vomiting, abdominal pain, and watery or bloody diarrhea^[^4^]^. Hemorrhagic manifestations include nose bleeds and vomiting, which were observed in five cases consistent with multi-organ failure^[^4^]^. The latest update as of last week shows the death toll has reached eight cessations along with 12 confirmed cases^[^6,7^]^. Considering the rising figures, 73 case patients were suspected of having MVD and tested, and 349 contacts were monitored^[^7^]^.

Nineteen outbreaks of MVD have been reported previously, but the most recent one was reported in Tanzania between January and March 2025^[^4^]^. This was effectively addressed by rapid outbreak detection and declaration, mobilization of response infrastructure, case management, and strengthening the health system post-outbreak. Hence, community engagement is the key to successfully controlling any outbreak. Outbreak control involves the use of a range of interventions, such as case management, surveillance, and contact tracing, for infection prevention.

Under the leadership of the Ministry of Health (Ethiopia), the WHO is working alongside Ethiopian response teams^[^8^]^ to enhance coordination and surveillance, including outbreak investigation, contact tracing, and alert management^[^4^]^. Home-to-home visits were minimized to reduce the risk of transmission through direct or close contact with an infected person^[^4^]^. Apart from the above-mentioned preventive measures, medical service delivery is being enhanced to swiftly and effectively tackle the unfortunate situation in the region. Strengthening cross-border surveillance, expanding rapid diagnostics, and enhancing community risk communication in local languages are urgently needed to curb further spread. This Correspondence is in line with the TITAN guidelines for the transparent reporting of artificial intelligence use in scholarly research ^[^9^]^.

## Data Availability

All data generated or analyzed during this study are included in this published article (and its supplementary files, if any).
